# A Microfluidic Dielectric Spectroscopy System for Characterization of Biological Cells in Physiological Media

**DOI:** 10.3390/s22020463

**Published:** 2022-01-08

**Authors:** Shide Bakhtiari, Mohammad K. D. Manshadi, Amin Mansoorifar, Ali Beskok

**Affiliations:** Mechanical Engineering Department, Southern Methodist University, Dallas, TX 75275, USA; sbakhtiari@smu.edu (S.B.); mdehghanmanshadi@mail.smu (M.K.D.M.); amansoorifar@smu.edu (A.M.)

**Keywords:** real-time cell characterization, electrode polarization, cell membrane capacitance, cytoplasm resistance, dendritic gold nanostructures

## Abstract

Dielectric spectroscopy (DS) is a promising cell screening method that can be used for diagnostic and drug discovery purposes. The primary challenge of using DS in physiological buffers is the electrode polarization (EP) that overwhelms the impedance signal within a large frequency range. These effects further amplify with the miniaturization of the measurement electrodes. In this study, we present a microfluidic system and the associated equivalent circuit models for real-time measurements of cell membrane capacitance and cytoplasm resistance in physiological buffers with 10 s increments. The current device captures several hundreds of biological cells in individual microwells through gravitational settling and measures the system’s impedance using microelectrodes covered with dendritic gold nanostructures. Using PC-3 cells (a highly metastatic prostate cancer cell line) suspended in cell growth media (CGM), we demonstrate stable measurements of cell membrane capacitance and cytoplasm resistance in the device for over 15 min. We also describe a consistent application of the equivalent circuit model, starting from the reference measurements used to determine the system parameters. The circuit model is tested using devices with varying dimensions, and the obtained cell parameters between different devices are nearly identical. Further analyses of the impedance data have shown that accurate cell membrane capacitance and cytoplasm resistance can be extracted using a limited number of measurements in the 5 MHz to 10 MHz range. This will potentially reduce the timescale required for real-time DS measurements below 1 s. Overall, the new microfluidic device can be used for the dielectric characterization of biological cells in physiological buffers for various cell screening applications.

## 1. Introduction

Cell screening is a method for observing living cells and evaluating cell physiology to discover cell properties and fate. This method has been widely employed for drug discovery [1,2], diagnostic purposes [3,4,5], biological molecules detection [4], and microbial species detection [6,7]. The traditional procedure for screening is based on microtiter plates, in which tests are conducted on 96-well plates. In order to reduce the cost and time of testing and enhance the efficiency, 384-, 1536-, and 9600-well plates were also introduced and employed for monitoring purposes. However, the drawbacks, such as evaporation, sample dispensing, and low amounts of samples, compelled the researchers to develop more efficient platforms [8]. Lab-on-chip (LOC) microfluidic devices provide the context to monitor cell functions in real-time using a small amount of samples with high levels of controllability. Various microfluidic platforms have been introduced by researchers for cell monitoring, such as continuous-flow-based screening, droplet-based screening, and dielectric spectroscopy (DS) based screening [8,9]. Among these methods, DS is a non-invasive, fast, and promising method for cell screening. DS has been widely used for impedance measurements of yeast cells [10,11,12], breast cancer cells [13], lymphocyte activation profiling [14], monitoring performance of epithelial tissues [15], and hepatology research [16].

DS-based cell screening often utilizes electrical impedance measurements of cell suspensions in the frequency domain. Cells’ dielectric properties, including cell membrane permittivity and cytoplasm conductivity, are extracted using various mathematical models, including the single-shell and double-shell models [17]. Other models for cells adhered to electrodes and single cells in flow cytometry also exist [18]. Reliable DS-based cell screening requires the minimization of cell–cell interactions, which were achieved by numerous methods, including the optical, acoustic, and thermophoretic tweezers [19,20,21], manipulation in viscoelastic fluids [22,23,24], and micro-well arrays [25].

Recently, our group presented a microfluidic DS device that captures individual cells in microwells using positive dielectrophoresis (DEP). This is followed by dielectric measurements and cell release using negative DEP [25,26,27]. The device allows dynamic alterations of the media so that the cell’s response to pH or osmolality variations or cell drug uptake can be measured in real-time. The ability of the device to release the cells after the measurements enable further biological studies [25]. Using this system, we investigated the responses of PC-3 cells (a highly metastatic prostate cancer cell line) to temporal pH variations [26] and the anticancer drug, Enzalutamide [27]. The primary limitation of our previous research was the necessity of using low conductivity buffers (LCB) to avoid the electrode polarization (EP) effects. The EP effects increase with increased buffer conductivity and reduced electrode size, and these overwhelm the impedance data in the β-dispersion regime, where charging of the cell membrane happens [25]. However, using LCB with conductivities that are ten folds lower than the typical cell growth media (CGM) has major consequences regarding cell shape regulation and pumping of ions out of the cells [26]. Therefore, an ideal microfluidic DS device should be able to avoid the EP effects, but at the same time, enable accurate measurements in physiological buffers, such as the CGM.

EP arises from charge accumulations on the electrode–electrolyte interface, which is due to the charge carrier prevention to cross the solid–liquid barrier. As a consequence, capacitance on the interface increases, which results in a significant electric potential drop along with impedance rise. EP has a considerable impact on microfluidic devices because of the small electrode surface area. An increase in the interfacial surface area is an effective way to reduce EP, which was achieved by electrodepositing dendritic gold nanostructures on the electrodes’ surfaces [28,29]. In this method, oxidation and reduction occur to deposit gold from a solution of gold ions onto the electrode surface. This reduction procedure generates repeated patterns with a fern-leaf-type self-similarity [30].

In this study, we demonstrate dielectric property measurements of PC-3 cells suspended in CGM as a function of time by using microelectrodes covered with dendritic gold nanostructures that minimize the EP effects. We also demonstrate a methodological approach for extracting the device and cell parameters from the equivalent circuit model. The reported cell membrane capacitance and cytoplasm resistance with 10 s increments show stable measurements for over 15 min, proving that the cells suspended in CGM were not affected by the microfluidic device and the dielectric measurements.

## 2. Materials and Methods

### 2.1. Device Fabrication

All chemical and photolithography supplies were purchased from Microchem Corp. (Westborough, MA, USA) and Sigma-Aldrich (St. Louis, MO, USA). To prepare all solutions, 18 MΩ·cm ultrapure water acquired from the Millipore Alpha-Q water system (Bedford, MA, USA) was used. To fabricate electrodes, first, the 2.5 cm × 2.5 cm glass slides were consecutively cleaned using an ultrasonic bath (FB11201, Fisher Scientific, Waltham, MA, USA) with 1 M KOH, acetone, isopropyl alcohol, and deionized (DI) water, for 10 min each at 25 °C and 37 kHz. Then, they were placed inside a 150 °C conventional oven to fully vaporize the water residues. The cleaned glass substrates were spin-coated with the positive photoresist (S1813). The rotation speeds of the spin-coater were 1000 rpm and 4000 rpm for 10 and 30 s, respectively, with 300 rpm/s acceleration/deceleration stages. After the substrates were soft baked for 1 min at 115 °C on a hot plate, and they were exposed to UV light through a transparency mask using a mask aligner (Karl Suss, MJB3) for 11 s. The substrates were submerged into a developer solution (MF-26A) for 17 s, and the UV exposed areas were washed away using DI water. Afterwards, the substrates were sequentially coated with chromium and gold layers for 60 mA-60 s setting using a sputter coater (EMS300TD, Emitech). The substrates were immersed into a PG remover to lift off the gold-coated photoresist regions.

The device in this study contains a pair of electrodes, of which only one has microwells on top of the electrode. To fabricate the microwells, a negative photoresist (SU8) was utilized on the 1 mm × 1 mm square shape gold electrode on the glass slide. An array of 21 × 21 microwells with 30 μm × 30 μm × 30 μm dimensions were formed on the sensing area. The dendritic nanostructures were fabricated on the bottom side of the microwells using the electrochemical deposition method in the optimal condition, as shown in our previous study [29], at −0.7 volt for 60 min. A three-electrode potentiostat/galvanostat system was utilized for electrochemical deposition (EZstatPro, Nuvant, Crown Point, IN, USA). Nanostructures were electrochemically built on the planar gold electrodes in 0.5 mg/mL Sodium Tetrachloroaurate (III) (AuCl_4_Na_2_H_2_O) solution (Sigma-Aldrich, Budapest, Hungary) using the deposition setup in the potentiostatic mode [18].

**Figure 1:**
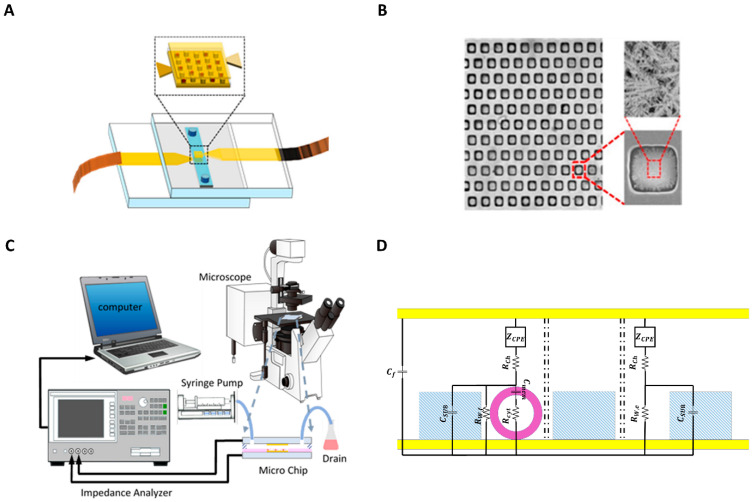
(**A**) Schematic of the microfluidic chip. (**B**) Picture of the microwells with nanostructured electrode surfaces. The figure shows the SEM images of the microwells array, zoomed view of a single microwell, and the dendritic structures on the electrodes on the bottom of the microwell. (**C**) Schematic view of the experimental setup. (**D**) Representation of the variables used in the equivalent circuit model of the device. The yellow part is the surface of the gold electrode. The purple is the membrane of the cell, and the blue part is the SU8.

The inlet and outlet fluidic holes were drilled on the glass slides using a diamond drill bit by a mini electric drill (Dremel 4000). A 70 μm thick double-sided tape was cut using a craft cutter (Silver Bullet) and used to make a microchannel. The microchannel was attached to the top electrode, and then the electrode pair were aligned using the mask aligner. The two electrodes were clamped to enhance adhesion between the slides, and copper tapes were connected to the electrodes for electrical connection. A schematic view of the microfluidic device is shown in Figure 1a, while the images of the microwells and SEM pictures showing the dendritic structures on the electrodes are shown in Figure 1b.

### 2.2. Experimental Setup

The experimental setup is shown in Figure 1c. The PC-3 cells with 10^5^ cells/mL concentration suspended in RPMI-1640 growth medium (Sigma-Aldrich) were injected into the channel from the inlet, and the cells were allowed to settle down in the wells for 5 min. This step was repeated three times, then the number of filled wells was counted under a microscope. To check the consistency of the gravity-based cell capture procedure, this method was applied to ten different devices. The mean value of the percentage of filled wells was 49.89% with ±2.60% standard deviation. The mean value corresponds to 220 filled wells out of 441 microwells. A syringe pump is used to supply growth media at a flow speed of 3 µL/min to wash the rest of the cells from the device. The cell suspension was collected from the outlet in a drain. The microfluidic chip was connected to the impedance analyzer (HP Agilent 4194A) to record the impedance spectrum. The impedance was measured in the 10 kHz–40 MHz frequency range at 20 mV AC voltage. Each impedance measurement sweep took six seconds for 314 discrete, logarithmically spaced frequencies, and the sweep was repeated every ten seconds. Then, the data from the impedance measurements were transferred to a PC and processed using a self-developed MATLAB code.

### 2.3. Cell Preparation

The PC-3 cancer cells were obtained from the Urology Lab at UT Southwestern, Dallas, Texas. The approximate diameter of the PC-3 cells is 22.0 ± 4 μm. The cells were cultured in RPMI 1640 supplemented with 5% fetal bovine serum (FBS), penicillin (100 IU/mL), and streptomycin (100 μg/mL). The cells were grown in an incubator (Thermo Scientific) at 37 °C with a 5% CO_2_ atmosphere. After 72 h, about 90% of the flask area was covered with cells, the growth medium was extracted, and the cells were washed with 1 × PBS, and 3 mL TrypLE was added to detach the cells from the surface. After incubating the cells with TrypLE for five minutes, 6 mL of complete growth medium was added, and the suspension was transferred to 15 mL centrifuge tubes. The cell suspension was centrifuged at 2000 rpm for four minutes, the supernatant was extracted, and a fresh medium was added to obtain 10^5^ cells/mL suspension for the experiments.

## 3. DS Method

DS is a method used to characterize the dielectric properties of biological cells by applying small AC voltage over a desired frequency range. The impedance can be obtained from the measured response current of an applied voltage. An equivalent circuit model with fundamental electrical components is used to analyze the impedance measurements, and the dielectric properties of cells are extracted from the impedance spectrum. Figure 1d shows the elements used for the equivalent circuit model for empty and filled microwells. We assume all EP effects to be identical for filled and empty wells, and these are characterized with the constant phase element model (CPE) with the impedance of Z_CPE_. EP is experienced at the electrolyte–electrode interface. Accumulation of charge at the interface causes large potential drop and low measurement sensitivity. This effect becomes dominant in the low-frequency range at high voltages and in highly conductive buffers. This unfavorable behavior of EP is modeled using the CPE model, defined as follows:

(1) $$Z_{\mathrm{CPE}}=\frac{1}{K{\left(j\omega \right)}^{\alpha }}$$

where α and K are the exponent and CPE coefficient, respectively. The angular frequency is denoted by ω, and j is the imaginary unit, where $j^{2}=-1$. The parameter α varies between 0 to 1, where 1 represents purely capacitive and 0 corresponds to purely resistive behavior. The value of K increases and α decreases with increasing the surface area [29].

Each filled channel has channel resistance, R_sol_, while each empty and filled well has its own corresponding resistance, R_w,e_ and R_w,f_, respectively. The microwells made out of SU8 are assumed to have capacitive behavior denoted with C_SU8_, while the biological cells in each filled well are modeled using the cell membrane capacitance, C_mem_*,* and cytoplasm resistance, R_cyt_. The stray capacitance in the entire system is shown with C_f_. It is important to note that each filled and empty well is assumed to be connected in parallel to each other. Details of this equivalent circuit model can be found in [26]. The total impedance can be written as:

(2) $$Z_{\mathrm{tot}}=\frac{1}{\frac{n_{f}}{Z_{f}}+\frac{n_{e}}{Z_{e}}+{j\omega C}_{f}}$$

where $Z_{e}$ and $Z_{f}\;$represent the impedance of empty and filled microwells, respectively, and n_f_ and n_e_ show the number of filled and empty microwells. Considering the equivalent circuit model, the impedance of empty microwells is written as:

(3) $$Z_{e}=Z_{\mathrm{CPE}}+R_{\mathrm{ch}}+\frac{1}{\frac{1}{R_{w,e}}+{j\omega C}_{\mathrm{SU}8}}$$

The filled microwell impedance is given as:

(4) $$Z_{f}=Z_{\mathrm{CPE}}+R_{\mathrm{ch}}+\frac{1}{\frac{1}{R_{w,f}}+{j\omega C}_{\mathrm{SU}8}+\frac{1}{\left(R_{\mathrm{cyt}}+\frac{1}{{j\omega C}_{\mathrm{mem}}}\right)}}$$

To calculate $C_{\mathrm{SU}8}$ as an SU8 capacitance, the following equation can be used:

(5) CSU8=εrε0ASU8t

where $\varepsilon_{r}$ is the relative permittivity of SU8, which is 3.2, and $A_{\mathrm{SU}8}$ and t are the SU8 surface area and SU8 thickness, respectively.

### Data Fitting Procedure

In order to find each electrical component from the circuit model and experimental data, real and imaginary parts of the impedance measurement were fitted to the real and imaginary parts of the equivalent circuit model using a nonlinear least-square method [26]. Overall, the following parameters are fit to the experimental data: $R_{\mathrm{sol}}$, $R_{w,e}$, $R_{w,f},K$, α, and $C_{f}$,${\;R}_{\mathrm{cyt}}$, and $C_{\mathrm{mem}}$.

The following procedure was used to find the unknown parameters, and an example of extracted parameters for each step is shown in Table 1. First, the impedance of the growth media (GM) was measured. Then, the nonlinear least square method was applied to the GM data to find the unknown electrical components: $R_{\mathrm{sol}}$, $R_{w,e}$, K, α, and $C_{f}$ (Table 1, Step 1). $R_{\mathrm{sol}}$ and $R_{w,e}$ represent solution resistance and resistance of the empty well, which should remain constant for both GM and CGM measurements. Thus, the boundaries of these two parameters were selected to be in the range of the GM in all data fits. After finding these values, PC-3 cells were injected into the wells, which resulted in filling approximately 220 wells, and the impedance of the system was measured. The curved fit was obtained, and all unknown parameters were calculated. It was observed that the new $R_{\mathrm{sol}}$_,_ $R_{w,e}$ from CGM were close to the values obtained for GM curve fitting, as expected before. However, due to the presence of cells in the wells, other parameters had different values compared with GM values (Table 1, Step 2). This curve fit procedure for CGM was performed for all time steps, which proved that K, α, and $C_{f}$ remained approximately constant through time (Table 1, Step 3). Therefore, we can conclude that impedance data changes through time were mostly related to the dielectric properties of cells ($R_{\mathrm{cyt}}$ and $C_{\mathrm{mem}}$).

## 4. Results and Discussion

**Figure 2:**
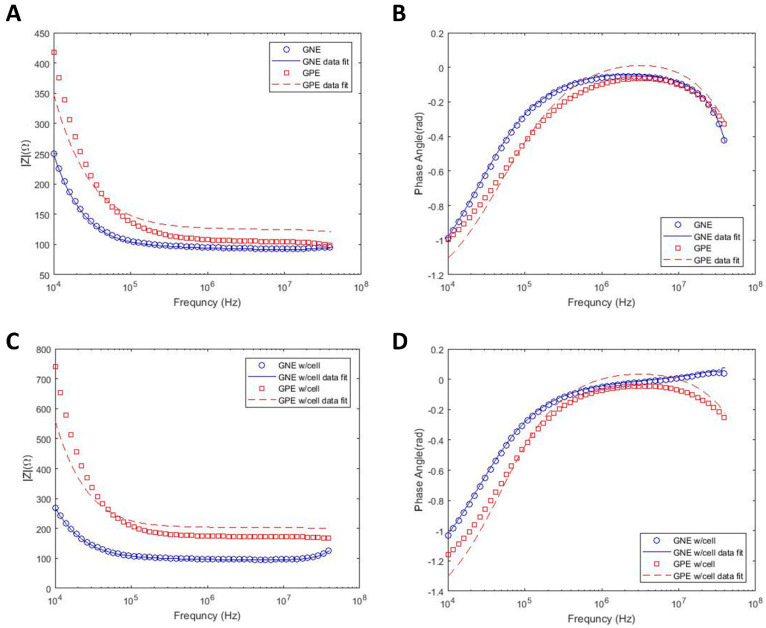
Impedance magnitude and phase angle spectra of GPE and GNE as a function of the frequency in growth media (RPMI-1640) with σ=1.5 S/m and curve fit with equivalent circuit model. Impedance spectra in growth media only (**A**,**B**) and in growth media with suspended PC3 cells (**C**,**D**).

DS measurements are usually performed in kHz to MHz frequency ranges, and the EP effect results in the reduction in measurement sensitivity in sub-MHz frequencies, especially in high conductive buffers, such as growth media. In this study, in order to decrease this undesirable effect that can overshadow the biological impedance response, the electrode–electrolyte interfacial area is increased by electrodepositing dendritic gold nanostructures on electrode surfaces on the bottom of the microwells, using three electrodes electrochemical deposition. Figure 1b shows an SEM image of the microwells array with a zoomed-in view of a single microwell and with details of the gold nanostructured electrode (GNE) morphology inside a well. In order to prove the benefits of the GNE electrode for the measurement of the biological cells, firstly, the impedance spectra of the growth media using both gold planar electrode (GPE) and GNE are measured, and the comparison is shown in Figure 2a,b. According to this figure, the measured impedance range for GPE with growth media is 420 (Ω) in 10 kHz and 100 (Ω) in 40 MHz. However, with electrode surface area modification, the impedance in 10 kHz frequency dropped to 250 (Ω), while it stayed the same as the GPE at 40 MHz. This clearly shows that GNE decreases the EP effect in sub-MHz frequency ranges and enhances the usable frequency bandwidth for biological measurements.

As mentioned before, in order to extract cell properties, such as membrane capacitance and cytoplasmic resistance, the experimental results are fitted into an equivalent circuit model using the data fitting methodology presented in the previous section. Lines in Figure 2a represent the fitted data on the experimental results for the device with growth media. However, the data fit error for GPE is higher than GNE (the R square for the corresponding fitted curve of GPE and GNE are approximately 0.8 and 0.99, respectively). The reason for data fit deviation from experimental results is the overshadowing of the impedance spectra due to the EP effect. Thus, the extracted parameters for GPE are not reliable. However, the results from the mathematical model fitted well on the experimental results of GNE due to the reduction in EP, which results in the extraction of acceptable parameters. In addition to the devices containing only growth media, the mathematical model is also applied to the acquired experimental data from GNE and GPE devices filled with PC-3 cells. Figure 2c demonstrates an accurate fit between model and experiment in device with GNE, which exhibits EP reduction. The extracted parameters from all graphs in Figure 2 are summarized in Table 2.

One of the important parameters in the equivalent circuit model is the number of filled vs empty microwells. The number of filled wells in the GNE devices could not be directly measured due to the opaqueness induced by the dendritic electrodes. As a result, the gravitational fill studies, as discussed in the Experimental Setup section, were performed using identical sized devices without the dendritic electrodes. Systematic experiments have shown that typically 220 micro-wells out of 441 were filled on average with a standard deviation of approximately ±11.5 wells. In order to verify the importance of the number of filled vs empty wells on the circuit model, we fit the circuit parameters for 220/441 (~50%), 233/441 (~53%), and 208/441 (~47%) fill ratios. The extracted model parameters are shown in Table 3. The effects of the well fill ratio on the cell membrane capacitance and cytoplasm resistance are 3% and 1%, respectively, while the filled well resistance varies less than 1%. These results prove the robustness of the equivalent circuit model.

**Figure 3:**
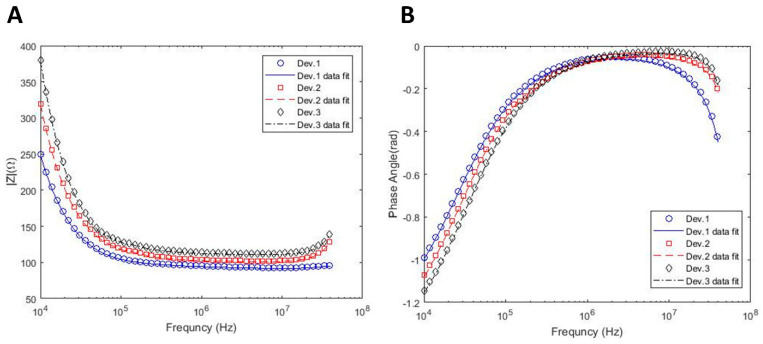
Effect of channel size on the impedance spectra within different frequencies. The widths of channels are 2.1, 1.7, and 1.4 mm for device 1, device 2, and device 3, respectively. (**A**) Impedance magnitude; (**B**) Phase angle.

One of the challenges in this study was the selection of channel width. Therefore, the effects of the channel width on the equivalent circuit model are also considered. Figure 3 shows the impedance spectra obtained from different sized channels with their corresponding curve fits based on the circuit model. The length of the channels is 15 mm for all devices, while the widths of channels are 2.1, 1.7, and 1.4 mm for device 1, device 2, and device 3, respectively. The extracted parameters from the fitted impedance curves are also summarized in Table 2. Cell properties such as membrane capacitance and cytoplasm resistance are merely independent of the channel width. Additionally, the solution resistance and resistance of empty wells from the three different channels are almost equal to each other, as expected from a consistent model. These results confirm that different channel sizes do not impose an effect on the extracted properties of the cells. This also shows the consistency of the electrical circuit model and the sequential data fitting procedure explained in the previous section. In addition to the cell properties, Table 2 also includes K and α values, which represent the CPE coefficients of interfacial impedance. According to this table, the electrode with a dendritic structure has a lower α (lower capacitance effect) compared with GNE, which shows DS measurement sensitivity enhancement in sub-MHz frequencies. However, GPE has higher α (higher capacitance effect) and lower K indicating the higher EP effect. It should also be noted that the values of K and α for the GPE electrode lacks accuracy, as shown in Figure 2. Moreover, by increasing the width of the channel, α decreased and K increased due to the reduction in the total impedance of the system.

**Figure 4:**
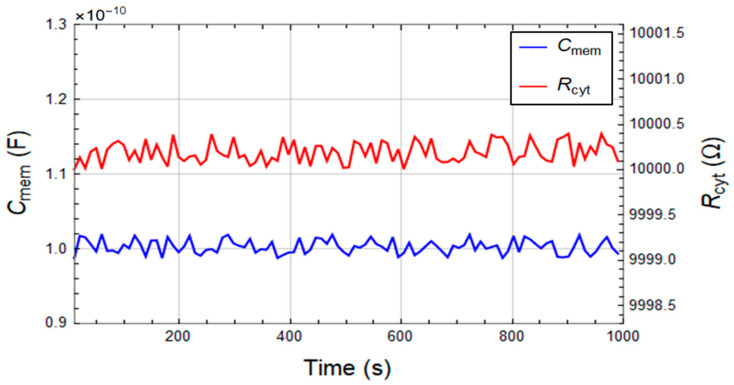
Time independence of the cell membrane capacitance (*C_mem_*) and cytoplasm resistance (R_cyt_) of PC-3 cells suspended in high conductive growth media (RPMI-1640) for 1000 s.

A significant characteristic of a promising microfluidic device for cell screening is its potential of maintaining the cells in a stable condition during the measurements. To verify this capability of the device, the membrane capacitance and cytoplasm resistance of PC-3 cells are measured every 10s for 1000 s while constantly feeding the system with high conductive growth media (RPMI-1640) at 37 °C. Figure 4 shows the stability of the extracted cell parameters as a function of time with C_mem_ = 100.30 ± 0.87 pF and R_cyt_ = 100.0020 ± 0.0001 kΩ. The results show that constant cell properties can be maintained in the device for over 16 min. We extended this period to 2.5 h and have not observed variations in the cell properties (data not shown for brevity).

The employed frequency range to extract cell properties in this study was 10 kHz−40 MHz. The fitted data in the previous figures and tables to find $R_{\mathrm{sol}},{\;R}_{w,e},R_{w,f},\;K,\;\alpha ,{\;C}_{f},{\;C}_{\mathrm{mem}},{\;\mathrm{and}\;R}_{\mathrm{cyt}}$ were calculated using 314 data points distributed logarithmically. In order to reduce the measurement time, the number of data points for property extraction was reduced to 88, 46, and 27 in the mathematical model. For these three data point numbers, the frequency ranges of 1MHz–10 MHz, 3MHz–10 MHz, and then 5 MHz–10 MHz were considered, respectively. The values of R_sol_, R_w,e_, K, α, C_f_ were obtained from the 10 kHz–10 MHz range of frequency using the 314 data points, and these parameters were assumed to be constants. Thereafter, $C_{\mathrm{mem}}{\;\mathrm{and}\;R}_{\mathrm{cyt}}$ values were found for the three mentioned frequency ranges. The results proved that using 314, 88, 46, and 27 data points to find the${\;R}_{w,f}$, $C_{\mathrm{mem}}{\;\mathrm{and}\;R}_{\mathrm{cyt}}\;$parameters led to the same results. As a consequence, a smaller range of frequency with a lower number of data points can be measured during the experiment to find the cell properties, which means lower measurement time and faster data analysis.

## 5. Conclusions

This study presents a microfluidic device and the associated mathematical models that can be used for real-time cell screening in high conductivity physiological buffers. Specifically, the dendritic gold nanostructures on electrodes reduce electrode polarization effects and allow the extraction of cell membrane capacitance and cytoplasm resistance. The systematic application of the equivalent circuit model for fitting the impedance data is demonstrated, and the measurements obtained at different times are correlated. Real-time impedance measurements taken with 10 s increments for 1000 s and data obtained from different width channels all resulted in the same cell parameters, which show the consistency of the circuit model and the ability of the device to maintain the cell properties for extended times. It has also been shown that the same cell parameters can be extracted using only 27 data points obtained in the 5 MHz–10 MHz range. This feature will reduce the real-time measurements below 1 s and will enable faster and easier data analysis. Future research focuses on the quantification of PC-3 cell response to various drugs and ion-channel blockers, while our long-term goal includes an extension of the current technique towards single−cell measurements, such that each cell trapped in a micro−well can be identified, sorted, and recovered after its response to external stimuli are investigated in a systematic fashion.

## Figures and Tables

**Table 1:** Sample of extracted parameters based on curve fitting on GM and CGM from the above procedure.

Step	R_sol_ (kΩ)	C_f_ (pF)	α	K	R_w,e_ (kΩ)	R_w,f_ (MΩ)	C_mem_ (pF)	R_cyt_ (kΩ)
1	27.432	6.03	0.71	2.59 × 10^−8^	16.329			
2	27.160	6.49	0.73	1.96 × 10^−8^	16.009	4.3279	99.95	10.000
3	27.149	6.49	0.73	1.96 × 10^−8^	16.007	4.3282	98.75	10.000

**Table 2:** Effect of channel width and nanostructure on the extracted parameters.

	Channel Width (mm)	R_sol_ (kΩ)	C_f_ (pF)	α	K	R_w,e_ (kΩ)	R_w,f_ (MΩ)	C_mem_ (pF)	R_cyt_ (kΩ)
Device 1 W/GPE	2.1	52.105	16.10	0.97	0.61 × 10^−11^	5.501	352.93	0.02	10.002
Device 1 W/GNE	2.1	27.160	6.49	0.73	1.96 × 10^−8^	16.009	4.328	99.95	10.000
Device 2 W/GNE	1.7	27.648	6.52	0.76	1.89 × 10^−8^	16.012	4.337	100.00	10.000
Device 3 W/GNE	1.4	28.281	6.48	0.78	1.84 × 10^−8^	16.004	4.291	99.97	10.000

**Table 3:** Effects of the standard deviation of filled well ratio on the extracted parameters.

Filled Wells	R_sol_ (kΩ)	C_f_	α	K	R_w,e_ (kΩ)	R_w,f_ (MΩ)	C_mem_ (pF)	R_cyt_ (kΩ)
47%	27.162	6.49 × 10^−12^	0.725	2.05 × 10^−8^	16.007	4.335	96.91	10.004
50%	27.160	6.49 × 10^−12^	0.729	1.96 × 10^−8^	16.009	4.328	99.95	10.000
53%	27.160	6.49 × 10^−12^	0.721	2.21 × 10^−8^	16.006	4.326	97.24	10.003

## Data Availability

Not applicable.
